# *QuickStats:* Age-Adjusted Death Rates* from Heart Disease^†^ Among Adults Aged 45–64 Years, by Urbanization Level^§^ and Sex — National Vital Statistics System, United States, 2019

**DOI:** 10.15585/mmwr.mm7046a8

**Published:** 2021-11-19

**Figure:**
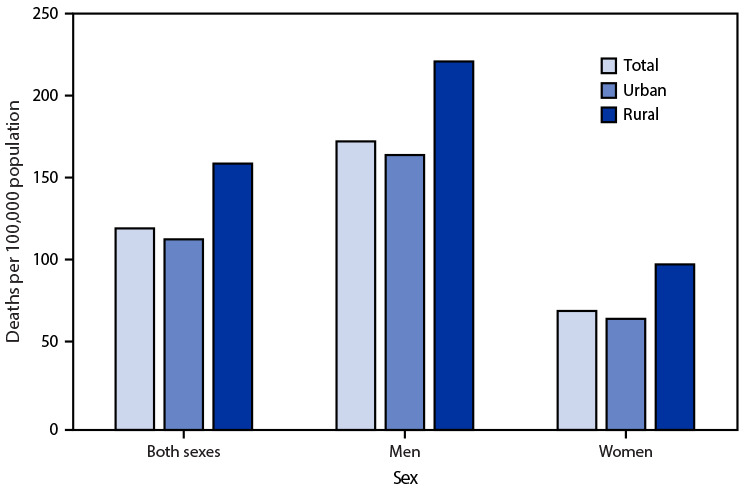
In 2019, the age-adjusted death rate from heart disease among adults aged 45–64 years was 121.1 per 100,000 and was higher in rural counties (160.0) than urban counties (114.5). Among men, the age-adjusted death rate from heart disease was 221.4 in rural counties and 165.1 in urban counties. Among women, the age-adjusted death rate from heart disease was 99.5 in rural counties and 66.8 in urban counties. In each urbanization level, the rate was higher for men than for women.

**Sources:** National Vital Statistics System, Mortality Data, 2019. https://www.cdc.gov/nchs/nvss/deaths.htm; CDC Wonder online database. https://wonder.cdc.gov/ucd-icd10.html

